# Barriers to Accessing and Engaging in HIV Preventive Care and Pre-Exposure Prophylaxis Experienced by Transgender Women in Florida

**DOI:** 10.3390/ijerph21030376

**Published:** 2024-03-21

**Authors:** Allysha C. Maragh-Bass, Sandra Kiplagat, Sarah Lavari, Francisco Sastre, Jessy G. Devieux, Daniel Jimenez, Rachel D. Clarke, Ines Noel, Eric W. Schrimshaw, Jae Sevelius, Elena Cyrus

**Affiliations:** 1 Global Health and Population Division, FHI 360, Durham, NC 27701, USA; 2 Department of Population Health Sciences, College of Medicine, University of Central Florida, Orlando, FL 32827, USA; 3 Robert Stempel College of Public Health and Social Work, Florida International University, Miami, FL 33199, USA; 4 Department of Prevention and Community Health, Milken Institute School of Public Health, George Washington University, Washington, DC 20052, USA; 5 Department of Psychology, Carlos Albizu University, Miami, FL 33172, USA; 6 Department of Medical Education, Herbert Wertheim College of Medicine, Florida International University, Miami, FL 33199, USA; 7 Department of Psychology, College of Arts and Science, New York University, New York City, NY 10003, USA; 8 Department of Prevention Science, University of California, San Francisco, CA 94158, USA; 9 Department of Psychiatry, Division of Gender, Health, and Sexuality, Irving Medical Center, Columbia University, New York, NY 10032, USA

**Keywords:** transgender women, Florida, HIV, PrEP, barriers to care

## Abstract

Background: PrEP, a biomedical HIV prevention option, continues to be underutilized among transgender women who could benefit from sustained use, especially women of color and those who identify as Latina and/or reside in the southeastern US. Objective: We explored the barriers and facilitators experienced by transgender women who live in Florida regarding accessing, using, and/or staying on PrEP. Methods: In-depth interviews and focus groups were conducted in either Spanish or English with adult transgender women living in Florida (N = 22). The interviews were audio-recorded, transcribed, and coded in ATLAS.ti using thematic analyses. Results: The mean age of the participants was 42.2 years. Among the participants, 73% were Hispanic/Latina, 59% were foreign-born, and approximately one-third were living with HIV (but had past experience with PrEP). Transgender women cited the following barriers to accessing or considering PrEP: (1) costs and benefits of PrEP use; (2) under-representation in clinical trials resulting in unknown or misinformation regarding PrEP side effects; (3) chronic poverty; and (4) trauma and discrimination. Other stressors, such as behavioral healthcare needs, were identified. Conclusions: Our analysis revealed interlocking systems of oppression like transphobia, discrimination, and misgendering, which were common barriers experienced by our participants. These synergistically epidemic (i.e., syndemic) barriers contributed to their feelings of being systematically excluded in social spaces, research, public health planning and policies, laws, and social programs related to PrEP. These structural barriers are impediments to HIV preventive care but also act as a source of stress that contributes to mental health problems, financial vulnerability, substance abuse, and other deleterious health outcomes.

## 1. Introduction

As of 2021, the global disease burden of HIV stands at approximately 38 million people, with 1.8 million new HIV infections acquired in 2021 [1]. In the same year, the United States (U.S.) observed an estimated increase of nearly 36,800 new HIV infections [2]. The southernmost tip of Florida continues to have a high incidence of HIV and is the home of many key at-risk populations, including U.S.-born and foreign-born transgender women immigrating from nearby Latin American and Caribbean regions [3,4,5,6]. In 2019, 25,651 people were living with HIV in Miami-Dade County, with 1194 new diagnoses in the same year, and the rates are estimated to have become higher since then [7].

HIV continues to represent a major public health concern for all racially minoritized women in South Florida [8,9], but there are limited data about the HIV inequities experienced by transgender women of color in this context. Some existing studies suggest that they have higher rates of HIV infection than cisgender men who have sex with men and other populations at high risk of HIV [8,9]. Compared to cisgender women of color, transgender women of color have an even higher risk of HIV infection; yet, they are often excluded from clinical trial research and surveillance studies, which limits the availability of accurate data about their needs and their access to the HIV preventive care that is designed with them in mind [2,8,10]. The limited available laboratory data documentation confirms that the HIV prevalence is approximately 14.1% for transgender women, compared to less than 0.5% for U.S. adults [10]. Additionally, a systematic review indicated that transgender women in the U.S. are 34 times more likely to live with HIV compared to the general population [11]. Therefore, it is critical to identify efficacious interventions among transgender women to improve HIV prevention and care.

Pre-exposure prophylaxis (PrEP) is a viable biomedical HIV prevention option for individuals at greater likelihood of being diagnosed with HIV, including but not limited to, transgender women who identify as Latina [12]. Despite this monumental advancement in HIV prevention (which is 99% effective when taken correctly), many transgender women remain unable to take full advantages of this therapeutic option for HIV prevention for reasons including inequitable care access [13]. Data from a 2019 study involving transgender women in South Florida show that while knowledge of PrEP was high in transgender women populations, the utilization of PrEP was as low as 8.2% [9]. In a 2020 study, researchers found that amongst Latina transgender women living in Los Angeles, both individual-level (e.g., lack of knowledge) and structural-level barriers (e.g., cost, access to gender-affirming care) led to PrEP discontinuation [14,15].

The underutilization of PrEP is one reason why there have been minimal changes in global HIV epidemiological trends for transgender women of color compared to other key populations. Other groups, such as cisgender White males, have consistently had greater access to care and have already experienced reverse trends and reductions in global HIV prevalence [13,14,15]. Beyond the barriers to accessing PrEP, other barriers transgender women of color experience include anticipated healthcare stigma and actualized structural-level stigmatization and intersectional discrimination uniquely experienced by those who are minoritized by both race and gender [15,16].

The existing literature on the unique experiences of transgender women of color seeking HIV preventive care in a South Florida context has numerous gaps; more research is needed to further (a) document their experiences and (b) characterize their intervention needs. Therefore, the objective of this study was to conduct exploratory research to understand facilitators and barriers towards the access and utilization of HIV prevention care—screening and PrEP—among transgender women living in South Florida. This study aims to explore the perceptions, systematic barriers, and experiences that prevent Floridian transgender women from accessing PrEP in order to document the common and specific barriers and to guide the development of future tailored HIV prevention interventions for transgender women.

### Conceptual Framework

To understand the lived experiences of transgender women, this study adopted the Socio-Ecological Model (SEM) and the theory of intersectionality as its conceptual frameworks [17,18]. The SEM provides an overview of the structural and social determinants of health (SDOH) that impact sexual risk behavior and decision making amongst transgender women, which consistently influence the odds or probability of HIV transmission [19]. The SEM considers how individual, relationship, community, and societal factors interact to affect health behaviors and risk factors. The theory serves to contextualize health behaviors through the lens of intrapersonal, interpersonal, community, and societal frameworks. Within the context of HIV acquisition, risk factors are present at all levels of the SEM [19].

Intersectionality offers a framework for understanding the interlocking systems of oppression experienced by people with multiple minoritized social identities [18]. As described by Crenshaw [18], intersectionality is the experience of systems of oppression, such as racism, sexism, and classism, and how they intersect to create disparities among people who are living at these intersections. In addition to the SEM, this study also utilized an intersectional approach to understand the varying systems of oppression that intersect to enable and reinforce perceived and experienced barriers to PrEP among transgender women in South Florida who may be racial/ethnic minorities and/or immigrants, because systems of oppression such as transphobia, misogyny, classism, racism, substance use, and sex work stigma will impact people’s ability to access PrEP; these barriers comprise synergistically epidemic barriers; therefore, interventions to address any of these barriers must address all of them to improve the outcomes among this population [20].

## 2. Methods

### 2.1. Study Setting and Study Population

The findings presented in this article are part of a larger mixed methods study that has the overall objective of adapting an evidence-based intervention that will ultimately assist transgender women with linking to substance use, PrEP, and other healthcare services. As part of this process, formative data were collected to guide the adaptation of the intervention, and these exploratory data regarding the perceived and experienced PrEP barriers are presented here.

Semi-structured in-depth interviews (IDIs) and three focus groups (FGs) were conducted among transgender women living in South Florida (N = 22) with any HIV status. All the women were asked about their life experiences as transgender women; however, transgender women living with HIV were asked questions in the context of linking to HIV care, and transgender women who were not living with HIV were asked questions related to linking to PrEP.

The study was conducted in and focused on the context of South Florida. South Florida primarily comprises the cities of Miami (Dade County) and Fort Lauderdale (Broward County). Compared to the rest of the state of Florida, this region is home to a much higher density of foreign-born residents, most of whom emigrated from the Latin American and Caribbean regions [19,21]. This is true of all immigrants in the region and is also reflected in the makeup of the majority of transgender women living in this community. Over two-thirds of the South Florida community identifies as Hispanic/Latine/Latinx, with many of these individuals also identifying as people of color and as foreign-born [20]. South Florida has also had one of the highest rates of HIV in the country since the beginning of the HIV epidemic; no reductions in HIV prevalence have been seen among communities of color in this region, including, but not limited to, transgender women of color who may also identify as Latina [21,22]. Moreover, the cost of living in South Florida is extremely high, which explains why the area has the third-highest rate of homelessness in the US [21,22].

We report findings from the analysis of eight in-depth individual interviews and three focus groups conducted with transgender women living in South Florida who were living with HIV (n = 14) or were HIV-negative (n = 8); thus, the total number of participants was 22. HIV-negative women who were not using PrEP discussed perceived barriers only (not current experiences), while transgender women who were living with HIV and HIV-negative transgender women who were already using PrEP reported on past and ongoing experiences and perceptions. The total number of interviews and focus groups conducted was the result of thematic saturation—the interviews and focus groups were conducted contemporaneously, and the preliminary findings were reviewed by study staff in real time to ensure that repetition in the comments was achieved prior to the completion of data collection [23,24].

The interviews and focus groups included a short questionnaire to collect general demographic information and substance use history. The interviews or focus groups ranged from 90 min to 2 h and were conducted in a private setting to ensure confidentiality. The interviews were conducted in the preferred language of the respondent (English or Spanish). For all the interviews, the participants selected preferred pseudonyms to protect their privacy.

The focus groups were conducted in English and translation support was provided for the non-English-speaking participants assigned to each group. To facilitate the focus groups with consideration of the sensitive nature of the questions, smaller focus groups were conducted (3 groups, each with 4 or 5 participants in total). Smaller groups allowed more candor among the participants and more time to allow the discussion to focus on aspects of experiences and needs that were the most salient and comfortable for the participants to describe (rather than focusing on addressing as many topics as possible). To further encourage candor, the groups were sampled according to HIV status (2 comprised HIV-negative women, while 1 focus group had only women living with HIV). The participants were invited to complete interviews or focus groups depending on whether the researchers felt they were more willing to share their experiences individually rather than with other women with whom they may have identified.

To explore key research topics, the IDIs and FGs were semi-structured and open-ended. The participants were asked questions regarding their health and other conditions related to syndemic factors of violence, substance use, PrEP engagement, and risky sexual behavior. For this manuscript and the present study, both the IDI (n = 8) and FG (n = 3; 14 respondents) responses regarding access to PrEP were analyzed, which allowed saturation on this theme around PrEP utilization. Both the interviews and the focus groups focused on the life domains (full life history) from childhood to adulthood development, family history, childhood trauma, and sexual history; however, the focus groups specifically explored adult experiences, including community norms, and experiences related to accessing and engagement in HIV preventive care. When relevant, the questions also explored women’s experiences with seeking HIV preventive care (since several were subsequently diagnosed with HIV even though they had previous experience with PrEP). Given the sensitive nature of both the interviews and the focus groups, the participants were reminded that (a) pseudonyms were going to be used for all analyses; (b) silence was okay and any questions could be skipped at any time; and (c) they could discontinue participation at any point.

### 2.2. Recruitment and Data Collection

Purposive sampling and participant referrals were used to identify participants, given that they are a population that is systematically under-recruited for clinical research and may be difficult to access as a result [22,23,24]. The potential participants were informed about the study by peer educators and navigators at the participating organizations. Additionally, the participants from a prior study conducted among transgender women at these organizations by the lead researcher were also contacted if permission had been documented [22]. The potential participants expressing interest were connected with project staff to assess eligibility and willingness to participate and to answer questions about the study. The interested participants were then screened via phone to determine eligibility. To be eligible, the individuals had to be adult (≥18 years) transgender women (assigned male at birth and currently identifying as female gender). Informed consent was obtained from all the participants before any study procedures were initiated and was obtained by the same study staff who conducted the interviews and focus groups to encourage comfort with the participants who may have less experience with participation in research studies (and for whom Spanish language participation was needed). The participants were then invited to refer to three other potential participants in the study. The participants recruited through the referral process followed the same screening protocol to determine study eligibility. Each participant received USD 50 gift cards for their time, regardless of whether or not they completed an interview or focus group. The study’s protocol was reviewed and approved by the institutional review boards of Florida International University and University of Central Florida (Study number: IRB Protocol Approval: #IRB-18-0046, IRB Approval Date: 16 January 2018, Study Reference: #106471).

### 2.3. Data Analysis

Data analysis was conducted in three stages: (1) exploration of recurrent themes and data integrity; (2) development of the codebook based on themes identified in the first phase; and (3) coding of the data using the codebook refined in the second phase. The data management, coding, and analysis were completed using ATLAS.ti software version 8.0 [25]. To maintain the integrity of the data, the interviews and focus groups were audio-recorded and transcribed by the research team using the participant’s original language. For the Spanish transcripts, the coding of the transcripts was completed in Spanish to match the original language of the interview. After the analysis, selected passages from the interviews and focus groups were translated into English for inclusion in the results. The data from the interviews and focus groups were exploratory in nature, and the sampling was not designed to compare and contrast (a) interview versus focus group modality or (b) experiences of women without HIV versus those living with HIV.

In the first phase, prior to initiating a systematic data analysis of the transcriptions, a precoding stage was performed by a coding team of four researchers from the study team (SK, FS, DJ, RC). This exploratory step included listening to the recording of each interview and focus group while reading the transcriptions to explore themes, in addition to verifying the quality of the transcriptions prior to commencing coding. At this stage, thematic patterns were identified in the data that informed the subsequent codebook development and data coding.

In the second phase, a preliminary codebook was developed by the coding team (with two levels of coding, i.e., parent and child codes). A section of a randomly selected interview (Fritz, part 3) was then coded to evaluate intercoder reliability and to discuss subsequent revisions to the codebook. This stage was repeated two more times with different transcripts (Fritz, part 1; Gloria) until an acceptable agreement of the intercoder reliability was reached (IRR alpha > 0.65 as per typical guidelines in qualitative research). A final codebook was developed as part of this multi-stage precoding process (Please see Table A1 in Appendix A).

During the third and final stage, transcripts were assigned to the research team members for coding. The transcriptions were coded according to the thematic categories that emerged from the data, i.e., the categories were developed, collected, and analyzed primarily from an inductive (with some deductive) thematic analysis perspective [22]. The codes described transgender women’s lived experiences and focused on the barriers and facilitators related to accessing HIV preventive care.

For the analyses in the present study, we focus on exploring the experiences of these transgender women with accessing, initiating, and adherence to HIV preventive care, including, but not limited to, PrEP. We focused our analyses primarily on the questions from the interview and discussion guides that pertained to barriers to accessing PrEP and staying on PrEP, along with the intervention needs specific to PrEP. The shared results and interpretations are kept as close as possible to the participants’ own words and are based on the understanding that their decision making is informed by their personal knowledge and their perceptions and experiences of the risks and benefits of accessing HIV preventive care. Due to the exploratory nature of our research and the small sample size, we did not conduct analyses by comparing women according to HIV status and/or racial/ethnic identities.

## 3. Results

The mean age of the participants was 45.6 (±14.6) years. More than half of the women self-identified as White; nearly 30 percent self-identified as being of more than one race; and just under 10 percent identified as Black. Over 70 percent self-identified as Hispanic/Latina, and nearly 60 percent of the total sample were foreign-born. Of the foreign-born population, one-fifth were born in Cuba, followed in order by Nicaragua (just under 15 percent). A similar number of individuals were born in El-Salvador, Honduras, Peru, Puerto Rico, and Uruguay (4.6 percent each; data on country of birth not presented in Table 1). Approximately one-third of the participants (31.8%) were transgender women living with HIV (Table 1).

Notably, a handful of participants identified as transgender women but were also involved in HIV preventive care provision themselves. Therefore, they were knowledgeable about speaking from the vantage point of their own lived experiences as well as about the barriers experienced by their clients and peers. Four themes pertaining to the lived experiences of transgender women of color related to HIV preventive care are described below and include (1) the costs and benefits of PrEP use; (2) under-representation in health research resulting in unknown information or misinformation regarding PrEP side effects; (3) chronic poverty; and (4) multi-level trauma and social exclusion.

### 3.1. Theme 1: Knowledge and Awareness in PrEP Use

Most of the participants (90.9%) reported knowledge and awareness of PrEP and its role in reducing the risk of HIV transmission; for a few, this was because they were involved in case management and PrEP navigation services in their own work. The participants mentioned that it was critical for PrEP to be provided in healthcare delivery spaces that provided wrap-around services incorporating PrEP medications, condom use, and HIV testing. While the participants had a high level of knowledge regarding PrEP use, they still had a major fear of contracting HIV.

*“It was more like I was freaking out because of- even though I take PrEP, and I do take precautions on protective sex, I’m like, “I hope I don’t catch it [HIV] and everything”—Liberty, FGD*

The participants expressed concerns that PrEP use may increase risky sexual behaviors among transgender women. Therefore, while PrEP is a groundbreaking medication to curb HIV, some participants voiced the concern that it may unintentionally and indirectly increase susceptibility to the contraction of other sexually transmitted infections (STIs) since PrEP only protects against HIV and not other STIs. Thus, some participants shared both experiences and perceptions related to the fact that PrEP cannot be an all-encompassing medication to prevent against all STIs outside of HIV and may further increase risk-taking behaviors among key populations.

To reduce these risks, some participants acknowledged the importance of utilizing condoms in protecting individuals from STIs and emphasized the point that condoms are effective in preventing STI transmission; therefore, it is critical to ensure that PrEP is used in conjunction with condoms to reduce the HIV/STI acquisition. Some participants also voiced their preference for condoms compared to PrEP since they perceived condoms as a double agent that reduces HIV and STI acquisition.

*“Ah, in the groups we always have…we always talk … I know about PrEP stuff we are talking always about the PrEP.—is one medication used to treat HIV people to treat that infection. But now, it can be also used for PrEP to prevent HIV, but only to prevent HIV not the otherIDs... So for me the better is the condom, the condom prevents everything.”—Gloria, IDI*

Some participants shared their experiences with transactional sex work, where some of their clients offer greater payment if the women were willing to engage in sexual behaviors which might increase their HIV risk, such as not using condoms. The women described feeling that they were being incentivized to not prioritize HIV prevention, which made it more difficult to prioritize other HIV preventive behaviors, such as using PrEP. This was highlighted in a focus group discussion:

*“And then I work with the population with sex workers and I found that even though sex workers we get informed, we get educated, we get on PrEP, but there’s still that barrier, that financial barrier where the client wants us to engage in high risk behavior. Because they’re [clients] not comfortable using condoms, they’re not comfortable with PrEP...”—Chardonnay, FGD*

Some clients may further encourage the use of post-exposure prophylaxis (PEP) compared to PrEP after engaging in sexual intercourse. One reason for engaging in PEP instead of PrEP is that PEP can be a single-point use after sexual contact with a client with less chance of any perceived weight gain that might occur with daily PrEP use. One participant who conducted case management work described the following:

*“For example, one girl told me, ‘oh, I had one of my doctors pay me a thousand dollars per session when I do it [sex] with no condom. And that client is a doctor. So, he knows everything… So, I said, oh OK, and take PrEP, right? [But after finding out about PEP, the girl was no longer willing to deal with PrEP because it’s so much more work. So], they forget about PrEP.”—Bella, FGD*

### 3.2. Theme 2: Under-Representation in Clinical Trials and Less Access to PrEP Information

In agreement with recent scientific findings/critiques, the participants described a lack of representation of transgender women in clinical trials. The majority of the clinical trial studies have focused on PrEP uptake among men who have sex with men, thereby excluding transgender women and other at-risk groups, ultimately resulting in limited evidence-based research study findings observed among transgender women. Specifically, one participant expressed the lack of long-term follow-ups in the studies in tracking adverse events among transgender individuals.

Given the scant research about PrEP medications among transgender individuals, there were some concerns expressed about the long-term side effects of PrEP medications among transgender women. Therefore, this needs to be considered a high priority for research scholars to ensure that PrEP studies intentionally engage and recruit transgender women in their studies so that the impact and adverse effects of PrEP use can be understood.

*“Because there is no specific study about [PrEP side effects that is specifically focused on transgender women] … it gives us so many complications with skin disease and then on top of that you have all these effects internally...”—Chardonnay, FGD*

More than half of the respondents (54.5%) stated they had had negative experiences with PrEP and that they had additional concerns about adverse effects, especially regarding medication/hormone interactions with PrEP use. In this sample, the adverse effects that were reported by the participants already using PrEP medication included: allergies, skin conditions such as rash and swelling, body odor, nausea, tiredness, and weight gain. While these side effects may be short-term, some participants cited concerns about unknown and long-term side effects associated with PrEP use, including those they had heard about from the clients they care for:

*“All the time when I refer girls to PrEP … they are gaining weight. And they‘re coming to me, oh but I am gaining weight [and getting skin problems] … And like for example, we referred 20 [girls] for PrEP In the last year and half, all but three cases came in with a lot of rashes”—Bella, FGD*

The participants expressed concerns that some of the experienced adverse effects had led to severe illness and hospitalizations. Due to these detrimental health outcomes, some of these individuals opted out of taking PrEP medication. Furthermore, some participants indicated their brand preferences between the two daily oral FDA-approved PrEP medications available at the time of the study, based on adverse effects associated with each of the medications.

Some participants, however, expressed negative perceptions of and hesitations about the newer approved medication, stating that it may not be 100% effective against HIV since it is relatively new to the market compared. Similar to medications which were previously FDA-approved, some participants described concerns about potential interactions and adverse effects of receiving both hormone and PrEP injections; thus, some of the participants reported opting to discontinue PrEP medication to prioritize hormone therapy. There was a concern among participants that using multiple strong therapeutics simultaneously (PrEP and medications associated with gender affirmation) could impact their long-term overall physical health:

*“Because a lot of the transgender [women] have not been in studies… long term they have found that they become more sick. They become more vulnerable, they feel more tired than what they [were before—but, did they eat healthy?] Have a healthy routine but they find themselves more tired? It’s like their organs is failing them. And then on top of that taking hormones is another drug supplement.”—Chardonnay, FGD*

### 3.3. Theme 3: Chronic Poverty and the “Cost” of PrEP

Chronic poverty, sustained throughout early childhood to adulthood, emerged as a barrier that prevented access to PrEP medication. This is in part driven by lifetime experiences of living in chronic poverty, including in pre-adulthood, as well as by the transient work opportunities among adult transgender women. Given the competing priorities in working and earning a living wage, PrEP was not considered a priority for some participants. Social determinants and basic safety were described as more important, in the form of affordable housing, food, and other basic needs. While refrigeration is not required for the medication, one participant highlighted how not having stable housing prevented the likelihood of staying on medication:

*“Sometimes we want to introduce them to PrEP, but we don’t see what their needs are. They don’t have a fridge to put the medicine [or sleep in the same place each night [or know if that is necessary for storing their medications] …”—Ivy, FGD*

Two participants highlighted the high cost of PrEP as a financial barrier when thinking of accessing PrEP medications and associated treatment. However, the perceived prohibitive cost may reflect a lack of awareness of existing programs and facilities where subsidized PrEP can be addressed. A myriad of contributing factors may be due to access, health insurance, misinformation about PrEP access, and the complexity involved in navigating the healthcare systems. Specifically, according to a focus group discussant, the client she was working with stated that there was a high copay for individuals enrolled in Affordable Care Act (Obamacare) who were interested in taking PrEP:

*“Um they have a copay, even the prep is free, but you have ObamaCare and the copay … it’s hard to pay like $220,”—Bella, FGD*

Although these departments’ PrEP programs provide free PrEP medication to those who qualify, not everyone was aware of this [26]. For example, a participant mentioned in the in-depth interviews that she met a young adult who was on PrEP who had been raped and had to pay USD 30 to access nPEP through her parent’s private insurance, which required a deductible. This out-of-pocket cost could have been prevented, however, given that HIV testing and PrEP access are free and can be accessible in the Florida Health Departments in Miami-Dade and Broward local offices. The participant informed the individual to go to the health department, and she was able to receive free services. Therefore, the lack of awareness and knowledge regarding accessing PrEP medication and engaging in consistent HIV prevention behaviors are barriers which impact transgender women in multiple ways:

*“… Listen… go to the health department and the health department was able to give it to her for free… [have] people out there to bring the message to the crowds, that’s how we can make the difference”—Theresa, IDI*

### 3.4. Theme 4: Multi-Level Trauma, Violence, Discrimination, and Healthcare Avoidance

Multiple participants described trauma beginning early on in childhood and continuing through adulthood, in the form of bullying and sexual assault perpetrated by peers, family members, and neighbors. This was often described as stemming from transphobia and discrimination. Reports of stigma and discrimination persisted from childhood to adulthood in different settings, including the home/residence, in schools before adulthood, in social settings, and/or in the workplace during adulthood. Most of the participants (85.7%) reported in the in-depth interviews that they had experienced stigma and discrimination from early formative stages, i.e., childhood and school age.

Women reported experiencing trauma in the form of physical assaults, during and/or after their gender transition and often when they were in social spaces with less racial, ethnic, and/or gender diversity in their adulthood. The participants reported that some acts of assault were committed by intimate and casual sexual partners:

*“Then after that I met my next boyfriend, which is my fourth partner and we dated for almost three years. We were in a committed relationship, we didn’t sleep with other people. But in the last year of our relationship, that was actually an abusive relationship for me. It kind of reminded me of back home when I was little. He abused alcohol a lot, so he hit me a couple of times and I would never fight back, because I loved him”—Versace, IDI*

The participants also reported experiencing violence perpetrated by law enforcement officers, further highlighting the structural and systemic violence the trans community is subjected to:

*“Me, three times he sufrido [I suffered] violence by some people in the street, including authority. Like police”—Lucero, FGD*

The participants reported experiencing multiple forms of stigma and discrimination from different public-facing actors, such as healthcare providers, law enforcement officers, and other LGBTQ community members, which discouraged them from any level of social engagement and resulted in healthcare avoidance. For example, one participant indicated:

*“Another thing is, many doctors say, I don’t treat people like you. I don’t understand what that means because I’m a human being…the problem is other people not educated enough to listen to us, and serve us”—Crystal, FGD*

Misgendering is the act of referring to or describing someone with pronouns that do not correctly align with their gender identity [27]. Misgendering can make it increasingly difficult for transgender women to navigate school and work settings successfully and is a reason multiple participants cited for why they avoid healthcare settings. Some of the participants felt unwelcomed and stigmatized for their transgender identity in the workplace, which may inadvertently result in social anxiety and mental health conditions. As a result, a participant reported presenting as male in work to avoid the misgendering or the constant attacks by coworkers:

*“At the same time, I still to this day even though I’m already living my life, because of certain things that tag me as a transgender woman and to avoid being misgendered, ridiculed or go through any those moments I don’t want to go through in my life, I still present at work as a male at my job still to this day. I don’t present as female out in my professional life, yet all my peers known I am a transgender woman,”—Gloria, IDI*

Several participants felt disempowered because the institutions that were supposed to serve as safe havens were the ones that damaged and harmed transgender individuals—examples include LGBTQ centers which mainly serve cisgender populations and often perpetuate transphobia inadvertently. Exclusion by other queer populations sustains the systematic exclusion of transgender women in key areas of society from informative data/surveillance research or public health planning, including the HIV preventive services that many LGBTQ centers may offer:

*“I know a lot of girls are very uncomfortable with the LGBTQ organizations that provides a lot of this [HIV testing and PrEP information] … I would say most girls don’t feel safe that I’ve come across in those types of environments because they are spaces which misrepresent and misappropriate us more than anyone. And a lot of girls don’t feel welcome, or safe and a lot of these environments or their spaces which are supposed to be safe for us”—Tawny, FGD*

Another example given by the participants was in law enforcement settings. While the duty of law enforcement officers is to serve and protect, some participants expressed concerns that they can also be included as perpetrators of stigmatizing and discriminatory behavior; they are not always seen as protectors, which reduced the participants’ willingness to access any institutions, including healthcare. One participant recalled being ridiculed and jeered at by a nurse in a doctor’s office during the registration and intake process:

*“So I remember going to a doctor some year ago. And when I walked in, I was nervous and scared how they were gonna treat me. And I walked in, and the nurse opened me and she looked at me, and told her I had an appointment. She handed me the board for me to fill out some papers. And when she shut the window, they all started cracking up laughing, they busted out in laughter. And I sat in the waiting room thinking how am I gonna trust these people with my healthcare if they’re looking me like a big joke,”—Sheila, FGD*

This treatment was often exacerbated for transgender women living with HIV since they were threatened by healthcare providers for not revealing their HIV status immediately. Some of these healthcare providers often indicated that they would report some of these individuals to law enforcement agencies. Therefore, there is a lot of distrust and disengagement with critical services such as PrEP that need to support the transgender population but often result in syndemic barriers which exclude transgender women from accessing and benefitting from HIV preventive care services [20].

### 3.5. Other Needs Impact HIV Preventive Care Engagement: Behavioral Healthcare

While these findings were not a major qualitative theme from our analysis, the participants described behavioral healthcare needs consistently in interviews and focus groups. Many women discussed societal stressors such as social marginalization and limited socio-economic opportunities, for which they needed coping mechanisms like alcohol and illicit drugs. The participants described mental health challenges that began early:

*“Um, it started all…when I was sixteen. It was right after the incident where I was raped and I got depressed to the point where I just wanted to die. Um, and I would constantly abuse pain killers, um, tons of laxatives, lots of weed and liquor, and stuff like that, and I was in a really, really dark place, and not even because of the fact that I was raped but also the fact that I was hating on myself because of what people would constantly call or talk about, about my body….”—Jean Gray, IDI*

## 4. Discussion

The purpose of the present research was to describe facilitators and barriers to HIV preventive care experienced by transgender women in a Southern Florida context, most of whom identified as Latina. This study contributes key insights about their needs, given that they are an under-represented population that is often excluded from health research though they continue to face some of the greatest HIV burden worldwide [2,8,10].

Although most of the sample are foreign-born and/or Latina, the experience of the participants in the study reflects the make-up of the larger South Floridian transgender community, which is primarily from the Latin American and Caribbean regions. From the conceptual perspective of the socio-ecological model (SEM), the existing research has documented the structural barriers to care often experienced by foreign-born populations, which are exacerbated by experiences of forms of discrimination due to (a) being transgender and/or (b) being of limited English proficiency and experiencing cultural barriers to adequate HIV preventive care [5,8,9,11].

Our participants described numerous instances of experiencing unmet needs related to HIV preventive care. From an intersectionality perspective, interlocking oppressive systems of racism, sexism, transphobia, and discrimination were described; these have resulted in multiple dimensions of reduced engagement in HIV preventive care for this population [28]: (1) lack of knowledge and information about PrEP; (2) under-representation of transgender women in clinical trials; (3) chronic poverty; and (4) trauma, violence, transphobia, and healthcare avoidance. Constant oppression due to these systems was also described in the form of coping with alcohol use and drug use; therefore, unmet behavioral healthcare needs were also identified and are an important priority for future interventions focused on transgender women of color in a South Florida context. The findings underscore the fact that their ability to remain in HIV preventive care is highly dependent on access to other vital forms of care and the intersectional context of the daily lived experiences in which they are seeking PrEP and other forms of HIV prevention [9,11].

Although our study participants acknowledged the role of PrEP in reducing HIV risk transmission consistently, there were still misconceptions about PrEP use, including the role of engaging in increased risky sexual practices and likelihood of HIV infection. Further education is needed to address misinformation and concern within the community, including pressure to engage in riskier behaviors when engaging in transactional sex work [13,29,30]. Much of the misinformation we heard from participants may reflect the lack of available clinical information given the consistent under-representation of transgender women in clinical trials. While this information can be considered to be missing or non-existent, there is also misinformation that is attributable to inaccurate information being dispelled in the local community due to this dearth. For example, many transgender women in the cohort expressed concern about the cost of care; however, for PrEP the state of Florida has at least subsidized the program rendering the prevention option as a cheaper or free option for most eligible women. PrEP is subsidized in the state of FL for the first three months of the use at least [26]. The confusion about the cost of and access to PrEP demonstrates the disconnect between public health policies promoting PrEP access and reality.

It is noteworthy that multiple participants reiterated their frustration with transgender women’s consistent under-representation in clinical trials, which may have been related to underlying concerns regarding side effects and interaction with gender-affirming hormone treatment. Prior studies have supported our findings that the consistent exclusion of transgender women in clinical trials can contribute to the decline in PrEP uptake which is now fueling a pervasive lack of information and/or misinformation regarding PrEP use in transgender communities [30,31]. Thus, it remains critical to include transgender women in clinical trials to benefit this key population. Relatedly, transgender women were less likely to opt for medical care, whether prevention or disease management, because they prioritized meeting basic needs or gender affirmation procedures over HIV prevention. This is particularly relevant as new administration modalities evolve for HIV prevention, including PrEP injectables and other manufactured options for the oral pill [32,33].

Our analysis revealed that experiences of transphobia, discrimination, and misgendering were the common experiences of our participants, which contributed to their feelings of being systematically excluded in social spaces, research, public health planning and policies, laws, and social programs related to PrEP (this exclusion is also related to intersectional oppression) [18]. These findings are consistent with those of previous studies that demonstrate that the systematic exclusion can be a major deterrent for transgender women seeking healthcare services [28,29]. Accurate information on PrEP may not be readily available at the healthcare provider level, or even at the community level, because at the community level fragmented social networks with scarce transgender-specific PrEP material resources engender distrust and apprehension around using PrEP. Specifically, there is a lack of information or there is misinformation about PrEP regarding potential adverse effects; drug interaction with other affirmative therapeutic procedures such as hormonal treatment; the cost; and how PrEP can be accessed in Florida without obstruction. Given the amalgamation of these factors, it is critical to ensure that health research in general, including, but not limited to, clinical trials and PrEP post-implementation studies [8,10,30,31,32,33], should be conducted among transgender women. Hence, healthcare providers can relay accurate information regarding PrEP medication among transgender women and address relevant concerns.

Among other key populations, such as men who have sex with men, with stronger social capital, misinformation/lack of resources can be mitigated by peer-level social capital [28,30]. A study in Peru highlighted the fact that it is critical to implement community-level responses to mitigate social exclusion and marginalization, ultimately to leverage social capital for transgender-specific social and cultural and community structures [28]. However, in Floridian transgender populations where there is greater division and social fragmentation, there is less chance of peer support supplementing for the structural gaps created by transphobic stigma and discrimination. Without any support at the structural level, the community level, or the family level, transgender women are forced to grapple with these situations in solitude while dealing with feelings of mistrust and insecurity and in some cases contemplating life-altering high-risk decisions (survival sex work, substance use, suicide ideation/attempts) that they believe can help to alleviate pain and confusion at a broader level.

While it was not a theme of our analysis, substance use and mental health issues were commonly described. Societal stigmatization of transgender women of color leads to syndemic outcomes of substance use and high-risk behavior, which can also act as barriers to care [34]. Syndemic issues are interconnected vulnerabilities that these women face, i.e., homelessness, substance abuse, violence, mental illness, and heightened HIV risk are both co-occurring and mutually reinforcing [35,36,37,38,39,40]. As an example, several women reported excessive substance use, and there were simultaneous reports of acts of violence, both of which are syndemic issues that have been shown to be prevalent within the transgender community [39]. Given the complex interaction of high levels of stigmatization, stress, substance use, and mental health issues, there is a need for culturally tailored interventions to address mental health to improve the behavioral health and the well-being among transgender women in Florida—future interventions must address all of these syndemic factors to see improvements in any outcomes, including, but not limited to, HIV preventive care uptake [37,38,39,40].

Similarly, at the individual level, cultural and social norms, systematic family and institutional discrimination, phobia, and misinformation were also major barriers. There is a saturation of PrEP public health messaging to transgender communities; however, the messaging seems to be lacking and missing the context of the lives of transgender women, with a considerable number of individual and structural barriers that must be overcome before they can have routine access to treatment or preventive medications. Prior studies have shown that transgender women are not also represented in the advertising and marketing messaging [29,33]. This is in part related to the under-representation of transgender women in both clinical and behavioral HIV research [37]. Given the critical need to seek, engage in, and adhere to PrEP services, there is a need to foster trust and patient-centered cultural sensitivity for transgender women in a variety of settings from healthcare providers, LGBTQ organizations, and law enforcement agencies.

## 5. Limitations

This study is not without its limitations. Given that the study was implemented just prior to the COVID-19 global pandemic, it is unclear how lived experiences in seeking care have changed for these women. The study also focused primarily on the experiences of transgender women who identify as Latina; while this is highly representative of South Florida, it limits the generalizability to other lived experiences and identities or other contexts in a state where foreign-born residents are fewer. While the study utilized purposive sampling [21], it captured variation in the sample in terms of place of birth, race/ethnicity, transgender identification, and length of time living as a woman, in addition to other common socioeconomic indicators, which helped to minimize the biases associated with this sampling approach [21]. Next, the data do not capture experiences with or perceptions of new injectables and recent marketing strategies which were not available at the time of this research; furthermore, our study focused mostly on access to PrEP, given the barriers experienced in this population, and speaks less to other HIV prevention modalities and/or to the needs of women already diagnosed and living with HIV.

## 6. Conclusions

PrEP engagement among transgender women is often a low health priority because of the syndemic barriers that these women face, including lack of knowledge and information about PrEP use, under-representation in clinical trials, chronic poverty, trauma, transphobia, intersectional discrimination, and other competing priorities like social determinants and behavioral healthcare needs. Given our narrow focus on the needs and experiences of transgender women who largely identified as Latina, future research should explore: (a) the experiences of transgender women from a broader range of racial/ethnic backgrounds; (b) HIV prevention modalities beyond PrEP (e.g., holistic care provision, including mental health support and community-based interventions); (c) longitudinal assessments of syndemic barriers to care and care outcomes in this population; and (d) greater accountability of community stakeholders regarding increasing the inclusion and representation of transgender women in clinical research and interventions. Points of intervention to improve access to care, including PrEP, should be culturally relevant and should simultaneously address misinformation through consistent accurate public health messaging and attempt to address the structural barriers that increase the individual’s vulnerability and risk.

## Figures and Tables

**Table 1:** Sociodemographic characteristics of adult transgender women living in Florida (n = 22).

	Proportions (n, %)
**Age (Mean ± SD)**	45.6 ± 14.6 years
**Race**	
Black	2 (9.1%)
White	12 (54.6%)
More than one race	6 (27.2%)
Other	2 (9.1%)
**Hispanic/x or Latina ethnicity**	
No	6 (27.3%)
Yes	16 (72.7%)
**Education**	
High school or GED or less	7 (31.8%)
College or higher	15 (68.2%)
**Monthly household income (US Dollars)**	
≤1000	7 (31.8%)
1000 ≤ 2000	7 (31.8%)
≥2000	8 (36.4%)
**Employment status**	
Employed for wages	8 (36.4%)
Out of work	4 (18.2%)
Self-employed	5 (22.7%)
Unable to work	1 (4.6%)
Retired	4 (18.2%)
**Country of Origin**	
U.S.-Born	9 (40.9%)
Foreign-Born	13 (59.1%)
**HIV Status**	
HIV-Negative	14 (63.6%)
HIV-Positive	7 (31.8%)
Refused	1 (4.6%)
**City**	
Miami	13 (59.1%)
Fort Lauderdale/Hollywood/Surrounding area ^a^	9 (40.9%)
**Length of residence**	
Less than 5 years	1 (4.6%)
1–5 years	3 (13.6%)
More than 5 years	18 (81.8%)

^a^ Surrounding areas may include Fort Lauderdale, Hollywood, Oakland Park, Pembroke Pines, Sunni Isles.

## Data Availability

The data presented in this study are available upon reasonable request from the corresponding author. The data are not publicly available due to sensitive subject matter and methodological concerns.

## Appendix A

**Table A1:** Qualitative codebook and key child codes used in qualitative analyses.

Parent Code: Agency	
*Advocacy (trans rights, services, community engagement, etc.)*	Definition/Context: Reported taking on the cause or seeking to defend the Trans community.
*Empowerment*	Definition/Context: Processes of becoming stronger and more confident, especially in controlling one’s life and claiming one’s rights; to act independently and to make free choices.
**Parent code: Cultural factors**	
*Gender/binary social guidelines*	Reported set of beliefs, moral values, traditions, language, and laws (or rules of behavior) commonly held regarding GENDER by a community.
*LGBTQ community*	Reported set of beliefs, moral values, traditions, language, and laws (or rules of behavior) commonly held by the LGBTQ community.
*Social status*	Reported set of beliefs, moral values, traditions, language, and laws (or rules of behavior) commonly held regarding social status or dictated by their social status.
**Parent code: Discrimination**	
*Law enforcement, court system, etc.*	An event or experience of unfair or prejudicial treatment based on characteristics such as race, gender, sexual orientation by personnel from any government entity.
*School/work/housing*	An event or experience of unfair or prejudicial treatment based on characteristics such as race, gender, age, or sexual orientation in an educational setting or work environment or in relation to housing (rent, purchase, mortgage, etc.).
*Services (health care, Trans-specific, community services, etc.)*	An event or experience of unfair or prejudicial treatment based on characteristics such as race, gender, age, or sexual orientation when seeking or receiving services in a health care setting.
**Parent code: Gender affirmation**	
*Acceptance*	Experiences related to receiving or experiencing acceptance and/or approval affirming cisgender and/or heterosexual identification.
*Lifestyle/language/passing*	Receiving or experiencing sexual advances that affirm cisgender and/or heterosexual identification.
*Transitioning*	Related to the process of transition that affirms cisgender and/or heterosexual identification—includes legal procedures (name change), non-surgery (therapy, etc.), and surgery.
**Parent code: Health behaviors**	
*Provider knowledge/competency*	Reported instance of health provider(s) demonstrating the lack or actual knowledge/competency about Trans health or issues while seeking health care. Reported instance of health provider(s) demonstrating the lack of actual sensitivity about Trans health or issues while seeking health care.
*Provider mistrust*	Reported suspicion or lack of trust in medical organizations, service provider, community organization for any reason.
*Sex (onset, history, etc.)*	Reported instance of sexually activity (oral, vaginal, anal, etc.) regardless of risk level.
**Parent code: HIV**	
*Beliefs*	Reported personal beliefs about HIV/AIDS, including general or specific FEARS about HIV/AIDS.
*Knowledge*	Reported knowledge and facts about HIV/AIDS, including reported MISINFORMATION or misconceptions about HIV/AIDS (facts, data, service location, etc.).
*Testing*	Reported testing or screening for HIV/AIDS.
**Parent code: Mental health**	
*Events (episodes, self-diagnosis, diagnosis trauma)*	Reported instances or experiencing of MH illness (episodes, self-diagnosed, or Dx, etc.), feelings of emotional or physical tension—all causes (stress) or trauma (any).
*Suicide*	Reported instances or experiences of suicide ideation, planning, contemplation, or attempt.
*Treatment*	Reported instances or experiences of receiving both traditional or non-traditional MH treatment (counseling, therapy, medication, residential, herb treatment, acupuncturist, etc.).
**Parent code: Misgendering/transphobia**	
*Health-seeking services*	Events or experiences of reporting being misgendered related to health care which may have resulted in healthcare avoidance (either HIV preventive care or otherwise).
*Misidentification* */transphobia*	Events or experiences of reporting being misgendered, including those based on legal documents, use of language, requests made that do not align with corresponding self-identified gender, etc.
**Parent code: Negative and adverse experiences**	
*Disapproval/rejection by family*	Reported instances of being rejected by family member or relative (all reasons, not just because of gender identification).
*Law enforcement, court systems, etc.*	Reported instances of negative experiences (not discrimination) by personnel from law enforcement, military, court system, or any government entity.
*Other (work, school, general)*	Reported instances of negative experiences (not discrimination) at school or work settings as well as any other experiences NOT captured by the other codes.
**Parent code: PrEP**	
*Barriers (cost, availability)*	Reported barriers to PrEP use, including ANY beliefs or perceptions about PrEP (access, use, side effects, etc.) that result in not accessing it.
*Facilitators (cost, availability, etc.).*	Reported factors to access, encourage, promote PrEP use, including incentives or benefits from or about PrEP (access, use, health, etc.), including ANY beliefs or perceptions expressed by respondent.
*Treatment/adherence* */side effects*	Reported treatment, adherence and side effects about PrEP (not perceptions or beliefs, but actual experiences reported by respondent or network).
**Parent code: Social determinants of health**	
*Homelessness/housing*	Reported instances of housing stability or housing insecurity. Code for all instances of homelessness.
*Immigration*	Reported instances of experiencing immigration (legal or undocumented). Use for documented immigration experiences (lifestyle).
*Race/racism*	Reported instances of experiencing acts or events of discrimination, abuse, prejudice because of race or ethnicity.
**Parent code: Support**	
*Community resources*	Reported instances of receiving services, support, encouragement, and genuine concern from community organizations (services, church, etc.).
*Family (chosen/origin)*	Reported instances of receiving empathy, compassion, encouragement, and genuine concern from a family member or relative.
*LGBTQ Community*	Reported instances of receiving empathy, compassion, encouragement, and genuine concern from another person(s) in the LGBTQ community.
**Parent code: Trauma/abuse/victimization**	
*Domestic/intimate partners violence*	An event or experience of violent or aggressive behavior within the home, typically involving the violent abuse of a spouse or partner.
*Emotional/sexual abuse in adulthood*	As an ADULT...any event or experiences of abuse or harassment that is emotional, physical, or sexual in nature. It can include anything from verbal abuse and constant criticism to more subtle tactics such as intimidation, manipulation, and refusal to ever be pleased; OR any event related to physical violence or harassment (use of physical force to hurt someone, physical attacks or threat, etc.).
*Law enforcement, court system, etc.*	Any event or experiences of abuse by personnel from law enforcement, within the court system, or any other government entity.
